# Design of Experiments Approach for Efficient Heavy Metals Stabilization Using Metakaolin-Based Geopolymers

**DOI:** 10.3390/molecules30153235

**Published:** 2025-08-01

**Authors:** Raffaele Emanuele Russo, Elisa Santoni, Martina Fattobene, Mattia Giovini, Francesco Genua, Cristina Leonelli, Isabella Lancellotti, Ana Herrero, Mario Berrettoni

**Affiliations:** 1 School of Science and Technology, Chemistry Division, University of Camerino, Via Madonna delle Carceri—ChIP, 62032 Camerino, MC, Italy; raffaele.russo@unicam.it (R.E.R.); elisa.santoni@unicam.it (E.S.); martina.fattobene@unicam.it (M.F.); 2 Department of Engineering “Enzo Ferrari”, University of Modena and Reggio Emilia, Via P. Vivarelli n. 10, 41125 Modena, MO, Italy; mattia.giovini@unimore.it (M.G.); francesco.genua@unimore.it (F.G.); cristina.leonelli@unimore.it (C.L.); 3 Department of Chemistry, Faculty of Sciences, Universidad de Burgos, Plaza Misael Bañuelos s/n, 09001 Burgos, Spain; aherrero@ubu.es

**Keywords:** alkali activation, metakaolin-based geopolymer, chromium salts, nickel salts, heavy metal stabilization, multivariate approach, design of experiments, principal component analysis

## Abstract

Alkali-activated aluminosilicate matrices are increasingly studied for their ability to stabilize hazardous metal contaminants via alkali activation at room temperature. In this study, metakaolin-based geopolymers were used to immobilize chromium and nickel salts, with systematic variation of key synthesis parameters, Na/Al molar ratio, metal concentration, anion type, and alkaline solution aging time, which have not been previously studied. A Design of Experiments approach was employed to study the effect of factors on metal leaching behavior and to better understand the underlying immobilization mechanisms. The analysis revealed that higher Na/Al ratios significantly enhance geopolymerization and reduce metal release, as supported by FTIR spectral shifts and decreased shoulder intensity. Notably, aging time had an influence on chromium behavior due to its effect on early silicate network formation, which can hinder the incorporation of chromium species. All tested formulations achieved metal immobilization rates of 98.8% or higher for both chromium and nickel. Overall, this study advances our understanding of geopolymer-based heavy metal immobilization.

## 1. Introduction

The present study originates from the need to address a specific waste stream, i.e., the galvanic sludges, characterized by an exceptionally high content of chromium (Cr) and nickel (Ni), which pose significant environmental and regulatory challenges due to their toxicity and potential mobility. The presence of high levels of chlorides and sulfates inhibits the stabilization/solidification (S/S) process using standard cement-based formulations (EN 1008; ASTM C 1602). To evaluate suitable treatment options for stabilizing this waste, alkali-activated materials were considered, including geopolymers (GPs) [1,2], due to their promising performance in heavy metal immobilization.

Geopolymers can effectively retain contaminants through chemical bonding or physical encapsulation within the matrix, as reported in the literature [3,4,5,6].

**Figure 1:**
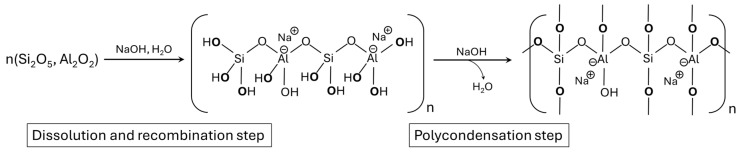
Schematic representation of the first and second steps of the formation of geopolymer materials.

Alkali-activated materials are an effective solution for the stabilization/solidification (S/S) of hazardous wastes, such as industrial sludges and contaminated residues [7,8]. They are obtained through alkali activation of aluminosilicate precursors, resulting in a three-dimensional inorganic network [9]. The process includes [10,11]: (i) dissolution of Si and Al species in alkaline conditions, forming –OH groups (Figure 1); (ii) condensation with water elimination and Na^+^ charge balancing near Al sites; and (iii) final polymerization into a rigid Si–O–Si and Si–O–Al matrix.

The resulting semi-crystalline framework, based on interconnected SiO_4_ and AlO_4_ tetrahedra, exhibits zeolite-like properties with intrinsic negative charges, which contribute to the immobilization of metal cations and other contaminants [12,13]. At the same time, the presence of short-range structural disorder within the aluminosilicate gel (NASH), facilitates ionic entrapment in nanostructured cavities [14,15], offer chemical environments similar to that of the zeolitic cages entrapping not only cations but also anions and even larger organic [14,16,17,18]. This complex nanostructure offers a wide range of S/S mechanisms within the same alkali-activated or geopolymer formulation [19]. S/S is a general term that indicates the reduction of the mobility and solubility of contaminants and pollutants, improvement of the waste handling and physical properties of the final product by creating a solid matrix free of free liquids, and minimization of the exposed surface area through which contaminant transfer or loss can occur [20].

“Solidification” refers to the incorporation of additives into the waste to form a solid material. This process may or may not involve chemical bonding between the contaminants and the matrix. “Stabilization” involves the transformation of waste into a more chemically stable form, typically through physicochemical reactions that reduce the contaminant’s mobility or toxicity. While stabilization may include solidification, it is primarily focused on altering the chemical nature of the contaminants [1,6,19].

These two processes often occur concurrently, driven by three main mechanisms: (1) chemical stabilization through the formation of insoluble metal compounds, (2) physical entrapment within the geopolymer matrix, and (3) electrostatic interactions between negatively charged aluminosilicate species and metal cations. Due to their simultaneous operation, it is often difficult to isolate the contribution of each mechanism [21].

In this work, a simplified model system was developed to study the maximum S/S capacity of metakaolin-based alkali-activated materials, often identified as geopolymers, for Cr and Ni, avoiding the complexity of real waste matrices. Two Cr(III) salts, chromium chloride and sulfate, and two Ni(II) salts, nickel chloride and sulfate, were selected due to their high solubility and representative anions. These anions were specifically chosen to investigate their influence on the stabilization mechanisms: chloride ions (Cl^−^) are known to promote the formation of sodalite-type structures by partially substituting for oxygen in the aluminosilicate network, potentially enhancing metal retention, whereas sulfate ions (SO_4_^2−^) are considered relatively inert due to their large steric hindrance and limited interaction with the geopolymer framework [9,22,23,24].

A Design of Experiments (DoE) approach was used to better understand how different formulation factors influence metal leaching from geopolymers. This method allowed us to study several parameters at the same time with fewer experiments. It helped identify the most important factors and their interactions [25,26]. The results were useful to understand the immobilization process and to improve the formulation for possible future use with real waste materials. In the literature, several studies have used Design of Experiments and mixture design approaches to optimize geopolymer formulations. Most of these works focus on the effect of different factors (e.g., curing and age conditions, alkali concentration, Si/Al ratio, and so on) by using mechanical properties, such as compressive strength, as the main response variables [27,28,29,30,31,32,33,34]. Other types of responses have also been investigated [35,36]. However, this work considers metal leaching as the response variable to study the effect of novel factors, such as the aging time of the activating solution, the type of metal salt, and therefore, the effect of the anion as well as the specific metal investigated. This approach also helps to propose possible immobilization mechanisms based on the observed leaching behavior.

In summary, this study advances previous research [37] by introducing and analyzing critical factors influencing the immobilization of heavy metals within geopolymeric matrices. The ultimate objective is to enable the effective stabilization of real waste materials containing high concentrations of these metals, ensuring their safe disposal and preventing potential environmental contamination.

## 2. Results and Discussion

### 2.1. Chromium and Nickel Quantification: Uptake and Leaching

Considering the different solubilities of the salts and the strongly alkaline formulations, the heavy metal content of the produced materials (Ni and Cr) was quantified according to the procedure outlined in Section 3.4. The effective concentrations of metal in each geopolymer formulation were quantified by ICP-OES following acid digestion. The effective metal content measured in the digested samples (columns 7 and 8) differs from the nominal content in column 6. Generally, when chloride salts were added, the difference between the nominal and effective contents was smaller (0.5–20%) with respect to sulfates (0.7–51%) considering both chromium (Cr) and nickel (Ni). Concerning the sulfates, when chromium (III) sulfate hydrate is added, the difference increases (22–38% for Cr vs. 0.7–20% for Ni) due to hygroscopicity, resulting in a lower content of the effective Cr with respect to the nominal. Further information on discrepancies between the nominal and effective metal content can be found in Table S4 (Supplementary Materials). The measured concentrations (columns 7 and 8) were used to update the design matrix, replacing the nominal values originally defined in the experimental design. Consequently, the mathematical model was fitted based on the measured Ni and Cr contents, ensuring that the response surface analysis reflects the real experimental conditions.

Table 1 reports the experimental metal concentrations (columns 7–8) along with their corresponding coded factor levels (columns 3–6). This reflects a modification of the original design, whichdid not result in a significant increase in multicollinearity, as the VIF (Variance Inflation Factor) values remained within an acceptable range from 1.00 to 3.09. The same table also presents the concentrations of nickel or chromium released in the eluate (leaching), which represent the response variables of the experimental design.

Table 1 also includes the assessment of geopolymer homogeneity, evaluated through the intrablock variability of two samples labelled “top” and “bottom”, corresponding to the upper and lower portions of the same geopolymer cubic specimen (*n*°.exp: 9, run: rep11). This approach allowed the investigation of internal heterogeneities within a single batch. The low variability observed between the two portions suggests that the geopolymer is macroscopically homogeneous under the conditions studied. Additionally, it was also observed that a low variance in the metal concentrations detected in the corresponding eluates, further confirming the homogeneity of the material.

### 2.2. Nickel

After running all the experiments and collecting the results, a multiple linear regression (MLR) was carried out:

(1) $$y=0.168+0.052x_{1}-0.125x_{2}-0.002x_{3}+0.125x_{4}-0.037x_{2}x_{3}-0.064x_{2}x_{4}+0.047x_{3}x_{4}+0.078x_{2}^{2}-0.049x_{3}^{2}+0.017x_{4}^{2}$$

The model was significant at a 0.05 significance level and did not have significant lack of linear fit at a 95% confidence level (*p*-value > 0.05). Residuals were randomly distributed and followed a normal distribution. Table 2 shows the coefficients and statistics of the fitted model, where the *p*-values less than 0.05 are the significant ones. Therefore, the model was valid and suitably explained the variability of the responses since the coefficients of determination is equal to 0.86; that is, 86% of the variability in nickel elution was described by the selected factors.

Moreover, the model was validated by prediction under the following experimental conditions: chloride was used as the anionic salt, the Na/Al ratio was set to 1.0, the initial nickel concentration was 1890 ppm, and the stirring time was 24 h. Under these conditions, the experimentally measured nickel concentration in the eluate falls within the model’s confidence interval, confirming the predictive reliability of the regression model.

Table 2 also shows that both the Na/Al ratio (x_2_) and the concentration of the eluate (x_4_) have a statistically significant influence on the response variable, which in this case, refers to the amount of nickel leached. In particular, the negative coefficient associated with the Na/Al ratio suggests that increasing this variable leads to a decrease in nickel recovery. Conversely, the positive and significant effect of eluate concentration is expected, as a higher initial amount of nickel in the sample naturally results in greater quantities detected in the leached solution of geopolymers. Table 2 also shows that chloride-based eluates yielded a higher amount of nickel compared to sulfate-based eluates (positive coefficient). This observation suggests that, despite not reaching statistical significance, the nature of the anion could still play a relevant role in the desorption mechanism.

The significant factors can be interpreted considering nickel’s chemical speciation under strongly alkaline conditions. In high-pH environments, such as those present in geopolymer systems, nickel tends to form insoluble basic salts, primarily as Ni(OH)_2_, which precipitate readily at pH values starting around 7 and remain stable even under highly alkaline conditions [38]. Therefore, it can be hypothesized that nickel is partially immobilized within the geopolymer matrix through precipitation mechanisms, rather than solely through physical encapsulation or chemical bonding with the aluminosilicate network. This can explain why only the initial nickel concentration and the Na/Al ratio emerged as statistically significant factors.

The nickel concentration factor determines the total amount of nickel available for leaching, while the Na/Al ratio affects the system’s alkalinity. The alkalinity of the activating solution enhances the first step of geopolymerization by increasing the SiO_4_ and AlO_4_ monomeric species, thereby affecting the second and third steps. As reported in the literature [27,39,40], increasing the alkali content enhances the overall degree of reaction, leading to a denser, mechanically stronger geopolymer matrix. Additionally, the higher alkalinity generated by the high Na/Al formulations promotes the precipitation of Ni(OH)_2_ and its retention within the geopolymer matrix [13].

**Figure 2:**
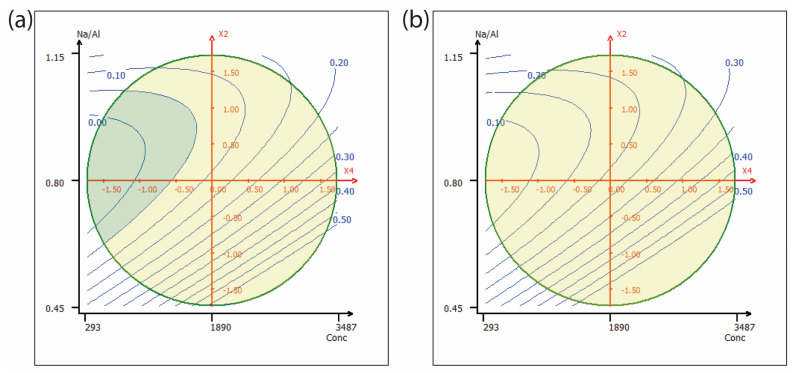
Response surface plots showing predicted nickel concentrations in the eluate as a function of the Na/Al ratio (x_2_) and metal concentration (x_4_) for (**a**) sulfate-based and (**b**) chloride-based systems. Other factors were kept at the central level. (yellow = below not-hazardous threshold, green = below inert waste threshold).

The response surfaces in Figure 2a, b illustrate the nickel leaching response across the experimental domain for sulfate and chloride salts, respectively. The green circle delineates the design space, while the contour lines represent the model-predicted concentrations of nickel in the eluate. The model indicates that nickel elution not only remains below the regulatory threshold for non-hazardous waste across the entire design space (yellow shape), but some concentrations fall below the inert waste threshold (green shape) in the case of sulfate-based eluates. This high immobilization rate was equal to or greater than 99.7% across all tested conditions as reported in Table 1.

### 2.3. Chrome

As done for nickel, once all experimental data were collected, a multiple linear regression (MLR) model was fitted to describe the chromium elution response. Prior to model fitting, the Studentized deleted residuals (R-student) were examined to identify potential outliers. As a result, two experimental points were removed due to their large residuals, which indicated inconsistency with the overall trend. Following this adjustment, the VIFs and the maximum leverage value (d_max_) were re-evaluated to verify that the model assumptions remained valid and that multicollinearity was still within acceptable limits. All VIF values remained below the commonly accepted threshold of 4, indicating that the estimated regression coefficients are statistically interpretable. Thus, the resulting equation is reported below:

(2) $$y=0.801+0.236x_{1}-0.456x_{2}-0.311x_{3}+0.228x_{4}-0.026x_{2}x_{3}-0.022x_{2}x_{4}+0.462x_{3}x_{4}-0.140x_{2}^{2}-0.089x_{3}^{2}-0.026x_{4}^{2}(*)$$

The model was statistically significant at the 0.05 level and showed no evidence of lack of fit at the 95% confidence level (*p*-values > 0.05). The residuals were randomly distributed and conformed to a normal distribution, confirming the validity of the underlying model assumptions. Table 3 shows the coefficients and statistics of the fitted model, where the *p*-values less than 0.05 are the significant ones. Overall, the model explained 93% of the total variance in the chromium leaching data.

As with the nickel model, the chromium model was also validated in prediction under the same experimental conditions. Also in this case, the result falls within the model’s confidence interval, further confirming its predictive reliability.

Table 3 also illustrates the magnitude and statistical significance of the regression coefficients obtained for chromium leaching. Among all the factors considered, several terms exhibited statistically significant effects on chromium leaching, as indicated in bold in Table 3. In particular, the type of anion, the Na/Al ratio, the aging time, and the interaction effect from concentration and aging time had significant contributions (*p* < 0.05). Although eluate concentration alone was not statistically significant (*p* = 0.07), its combined effect with aging time was significant, indicating a relevant interaction between the two factors.

Compared to the nickel model, the only notable difference lies in the aging time and in the anion type, which are statistically significant in the chromium model. In fact, the positive coefficient of the anion type means that chloride-based eluates result in considerably higher chromium release than sulfate-based ones. From literature data [14], a possible explanation for the chloride salts can be found in its repulsion by the negatively charged silicates surface especially where non-bridging oxygens are localized. The smaller size of Cl^−^ ions leads to a higher charge density with respect to sulfate ions exhibiting a weaker adsorption and lower stability of the cation–anion ionic pair near the interface [14].

To explain the significance of aging time, the chemical behavior of chromium under alkaline conditions was considered. Cr^3+^ generally precipitates as Cr(OH)_3_ in the pH range of 6–12. However, in the presence of excess alkali, it forms soluble anionic species, such as CrO_2_^−^ and other moieties [38]. This aspect is particularly relevant in light of the experimental findings, which show that both the aging time of the activating solution and its interaction with metal concentration are statistically significant factors. The interaction term is positive, indicating that longer aging times combined with higher chromium concentrations result in increased leaching.

To the best of our knowledge, no literature is available on the role of activating solution aging time on chromium (or any other metals) release from geopolymer matrices. From a structural perspective, Cr^3+^ is known to adopt an octahedral coordination environment (CrO_6_), and it has been proposed that chromium could chemically interact with the three-dimensional aluminosilicate network, forming Cr–O–Al bridges rather than segregating as pure oxide phases, such as Cr_2_O_3_ [17]. It is possible to hypothesize that longer aging times between sodium hydroxide and sodium silicate could lead to the early formation of silicate frameworks, reducing the ability of the geopolymer network to effectively incorporate chromium species. Further investigations will be carried out to confirm this hypothesis.

**Figure 3:**
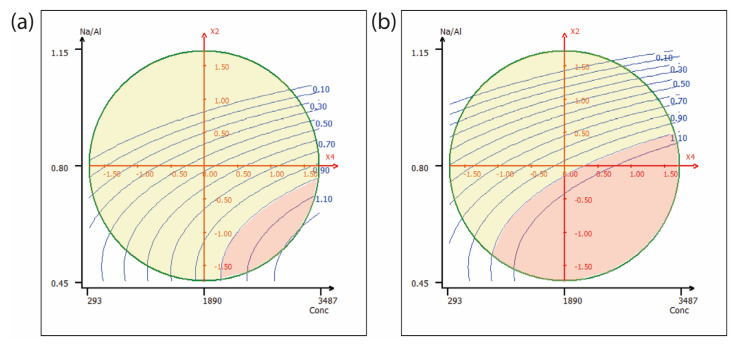
Response surface plots showing predicted chromium concentrations in the eluate as a function of the Na/Al ratio (x_2_) and metal concentration (x_4_) for (**a**) sulfate-based and (**b**) chloride-based systems. Other factors were kept at the central level. (yellow = below not-hazardous threshold, red = hazardous waste).

Figure 3 shows the response surfaces for chromium elution as a function of the Na/Al ratio (x_2_) and eluate concentration (x_4_). Figure 3a corresponds to sulfate-based eluates, while Figure 3b refers to chloride-based conditions. As with nickel, the yellow regions correspond to conditions classified as non-hazardous waste, while the red regions indicate hazardous waste. No areas within the design space fall below the threshold required for classification as inert. As for the immobilization rate, all tested conditions showed values equal to or greater than 98.8%, as reported in Table 1.

### 2.4. FTIR Analysis

**Figure 4:**
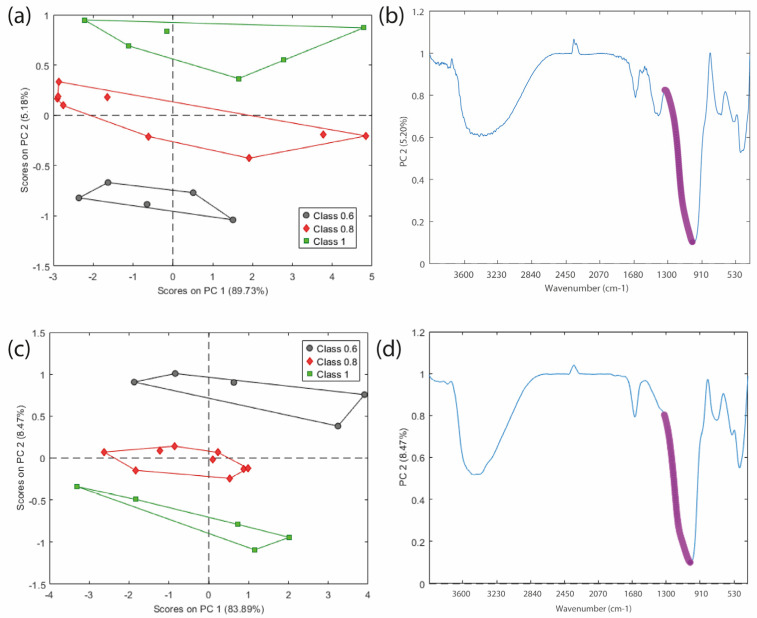
PCA applied to FTIR spectra of geopolymer samples. Panels (**a**,**b**) show the score plot and corresponding loading plot for the Ni-based systems, while panels (**c**,**d**) show the score and loading plots for the Cr-based systems.

The Principal Component Analysis (PCA) score plot for nickel and chrome in Figure 4a–c shows a clear separation of the samples according to their Na/Al ratios, confirming that this compositional variable has a significant impact on the material as shown in Table 2 and Table 3 (*p*-values < 0.05). The second principal component (PC2) is particularly effective in differentiating the samples, suggesting that spectral variations correlate with the Na/Al molar ratio.

**Figure 5:**
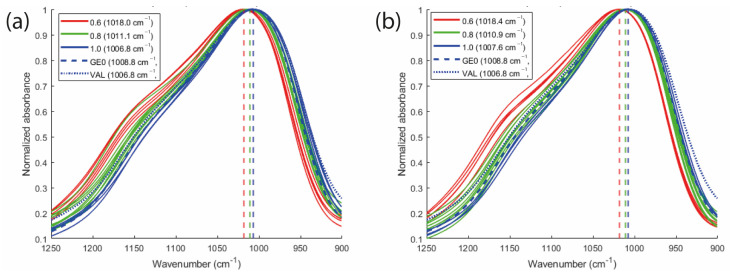
FTIR spectra (1250–900 cm^−1^) of geopolymer samples with different Na/Al molar ratios (0.6, 0.8, 1.0) for (**a**) Ni and (**b**) Cr systems. GE0 is the geopolymer without metals; VAL is the validation experiment with Na/Al molar ratio equal to 1.

Figure 4b–d presents a post-processed visualization of the average spectra, where the violet points indicate, respectively, the negative and positive loadings of PC2. These spectral variables correspond to the shoulder observed in the peak region between 1090 cm^−1^ and 1180 cm^−1^, which contributes most to the separation of the three Na/Al groups observed in the corresponding score plots. According to literature studies based on spectral deconvolution [41], this shoulder arises from Si–O stretching around 1095 cm^−1^, Si-O-T (where T = Si or Al) asymmetric stretching around 1070–1080 and Si-O-Si asymmetric stretching vibrations occurring between 1110 and 1140 cm^−1^. In this study, for both chromium- and nickel systems, a clear trend was observed: as the Na/Al ratio decreases, the intensity of the shoulder increases, whereas higher Na/Al ratios result in its reduction (Figure 5). This behavior can be attributed to the role of Na^+^ ions in charge balancing. In geopolymer systems, the incorporation of aluminum into the silicate network introduces a negative charge that must be compensated by alkali cations, primarily Na^+^, K^+^, or Ca^2+^. At low Na/Al ratios (e.g., 0.6), the alkali content may be insufficient to fully balance the framework charge, leading to incomplete geopolymerization and a higher proportion of unreacted Si–O–Si species. This results in a more pronounced spectral shoulder, reflecting the presence of less cross-linked silicate structures.

Figure 5 also shows a clear shift of the main infrared band in both metal-containing systems, driven by changes in the Na/Al ratio. This band, as mentioned above, corresponds to Si–O–T asymmetric stretching vibrations and is a key indicator of geopolymerization. In metakaolin, it typically appears around 1060–1100 cm^−1^. After alkali activation, it shifts to lower wavenumbers, indicating the incorporation of Al into the silicate network and the formation of the geopolymeric structure [42,43,44].

Overall, both the decreased shoulder intensity and the progressive shift of the main Si–O–T band toward lower wavenumbers highlight the strong influence of the Na/Al ratio on the geopolymer structure. These spectral changes suggest a gradual structural reorganization of the aluminosilicate network, promoted by a higher alkali content (Na/Al = 1). Thus, this compositional parameter, therefore, plays a dual role: it not only controls the evolution of the geopolymerisation but also the efficiency of metal immobilization, as demonstrated by the DoE results.

### 2.5. XRD Analysis

**Figure 6:**
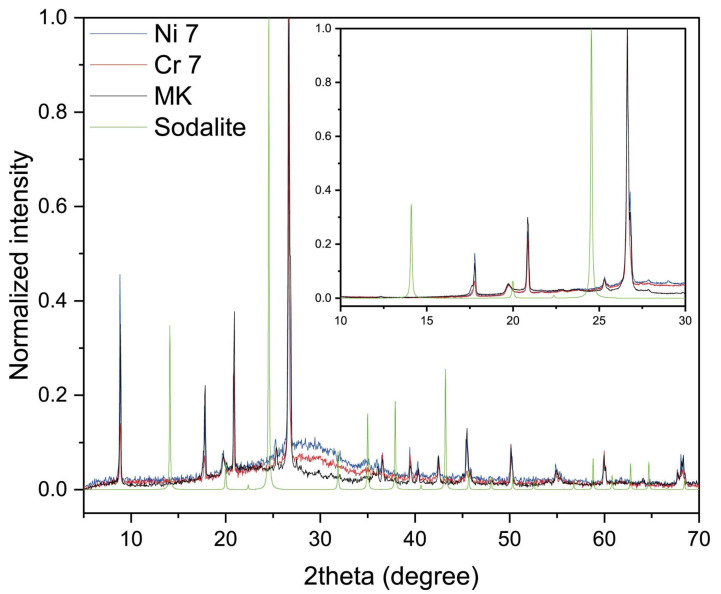
XRD patterns of metakaolin (MK) and geopolymer samples with nickel (Ni 7) and chromium (Cr 7). The inset highlights the 10–30° 2θ region where sodalite peaks are expected.

X-ray diffraction (XRD) analysis was performed to verify the formation of sodalite or other zeolites, using the Crystallography Open Database (COD) reference 1,000,028 for sodalite, with the crystal structure Na_4_(Si_3_Al_3_)O_12_Cl [45]. For this purpose, the samples with a higher chloride content, specifically those prepared using formulation 7 for both Ni and Cr systems (Na/Al ratio = 0.8, 15 h, 3450 ppm Cr), were selected for analysis. The sample was collected using a PMMA specimen holder, 25 × 8.5 mm. These samples were then compared with both the sodalite reference pattern and the original metakaolin (MK) starting material (Figure 6).

As shown in the diffractograms, both in the full 2θ range scan and in the focused region between 10° and 30°, which includes the main sodalite reflections (green bars), all diffraction peaks correspond to the metakaolin. The only noticeable difference in the pattern of the MK compared to the two geopolymers is the presence of a broad amorphous phase, which is typical of an N-A-S-H gel [46]. The amorphous halo shifts from 20–30° in 2θ for MK to 25–35° for the geopolymers as the insertion of the Al tetrahedral units distorts the silica disordered network [47].

No additional peaks attributable to sodalite are detected, confirming that sodalite did not form under the investigated conditions, according to what is reported in the literature, where a higher thermal level is necessary [48,49,50]. Nonetheless, its presence cannot be definitively excluded, as can be present in concentrations close to the detection limit of the technique.

## 3. Materials and Methods

### 3.1. Reagents and Materials

In this study, ARGICAL™ M1000 metakaolin (MK) (sourced from Imerys, Paris, France) was used. According to the manufacturer’s data, the chemical composition of this MK is as follows: SiO_2_ = 55 wt%, Al_2_O_3_ = 40 wt%, Fe_2_O_3_ = 1.4 wt%, TiO_2_ = 1.5 wt%, Na_2_O + K_2_O = 0.8 wt%, CaO + MgO = 0.3 wt%, and LOI (Loss on Ignition) = 1 wt%. NaOH laboratory-grade granules (96 wt%, Sigma Aldrich, Milan, Italy), in combination with a commercial sodium silicate solution (Ingessil, Verona, Italy) with a SiO_2_/Na_2_O molar ratio of 3.0, containing 26.50 wt% SiO_2_ and 8.70 wt% Na_2_O, pH of 11.7 and density of 1.368 g/cm^3^ at 20 °C, was used in the formulation of the geopolymers. The salts introduced into the geopolymer matrix as synthetic waste were chromium (III) chloride hexahydrate (98 wt%, Sigma Aldrich, Milan, Italy), chromium (III) sulfate hydrate (Cr_2_(SO_4_)_3_ · xH_2_O, x = 0 to 18 mol, Sigma Aldrich, Milan Italy), nickel (II) chloride hexahydrate (98 wt%, Merck, Milan, Italy), and nickel (II) sulfate hexahydrate (98 wt%, Carlo Erba, Milan, Italy). TGA analysis estimated the chromium (III) sulfate hydrate to be epta-hydrate, although we could not rule out significant and difficult-to-estimate hygroscopicity.

### 3.2. Geopolymers Formulation

**Figure 7:**
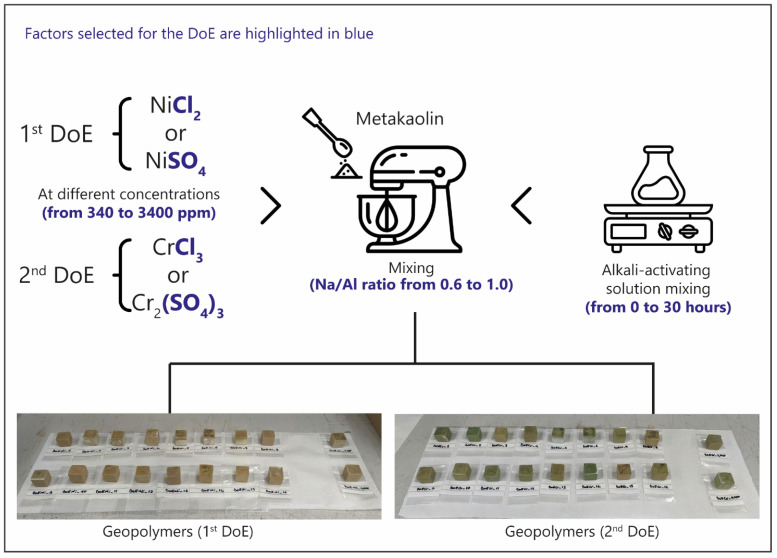
Schematic representation of the experimental design strategy used to formulate geopolymers for heavy metal stabilization. The 1st DoE is refers to Ni salts while the 2nd to Cr salts.

The procedure used for sample preparation, schematically illustrated in Figure 7, was developed based on previous studies [17,51]. Initially, the activating solution was prepared by mixing sodium hydroxide pellets with sodium silicate for a predetermined time (0–30 h), according to the variables defined in the Design of Experiments (DoE).

The amount of sodium silicate was fixed at 34 mL, while NaOH was varied (4.12, 7.32, and 10.52 g) to achieve Na/Al molar ratios of 0.6, 0.8, and 1.0 in the geopolymer formulations.

Subsequently, the activating solution was mixed with metakaolin for 5 min to initiate the alkaline activation of the aluminosilicate raw material. At the end of this period, chromium and nickel salts, previously dissolved in 15 mL of distilled water, were added to the geopolymer paste and mixed for an additional 5 min to ensure effective incorporation. Any air bubbles entrapped in the paste were then removed using a vibrating table. The resulting mixture was poured into silicone cuboid molds and left to cure for 48 h in a sealed container. Once solidified, the samples were demolded and left to cure in ambient air until further analysis.

### 3.3. Chemometric Approach

An experimental design (DoE) approach was employed to investigate the stabilization performance of metakaolin-based geopolymers with respect to the leaching behavior of heavy metals. The study focused on the release of chromium and nickel, assessed according to standard leaching test protocols, which was used as the main response variable. Two separate circumscribed central composite (CCC) designs were developed: one for chromium and one for nickel, allowing for an independent evaluation of the factors affecting the immobilization of each metal.

The investigated factors (Table 4) included the type of anionic environment (chloride or sulfate); the initial concentration of the target metal (340–3440 ppm); the aging time of the alkaline activating solution (0–30 h); and the Na/Al molar ratio (0.6–1.0). The experimental domain was selected based on prior experience and literature [7,52,53,54]. The initial concentration of the target metal was varied between 340 ppm and 3440 ppm. The lower limit corresponds to the maximum Cr^3+^ concentration that, according to the literature [7], can be fully immobilized within the geopolymer matrix under optimal conditions. The upper limit was chosen arbitrarily as a tenfold increase in order to evaluate the material’s retention capacity under more challenging loading conditions. The aging time of the alkaline solution ranged from 0 to 30 h and refers to the time interval between the mixing of the sodium hydroxide (NaOH) with the sodium silicate solution (Na_2_SiO_3_, R3) and its subsequent addition to the metakaolin. Figure 7 shows clearly the experimental factors.

Finally, the Na/Al molar ratio was varied between 0.6 and 1.0 based on previous studies in the literature [52,53,54]. Higher Na/Al molar ratios were not considered, as the addition of NaOH pellets to increase the sodium hydroxide concentration in the activating solution led to solidification when the Na_2_O/SiO_2_ molar ratio exceeded 1.0, making the solution unworkable.

The experimental design methodology enabled the analysis of main, interaction, and quadratic effects, aiming to optimize the geopolymer formulations for enhanced immobilization efficiency. Thus, the postulated multiple linear regression model is composed of 11 terms (1 constant, 4 linear terms, 3 interactions, and 3 quadratic terms):

(3) $$y=b_{0}+b_{1}x_{1}+b_{2}x_{2}+b_{3}x_{3}+b_{4}x_{4}+b_{23}x_{2}x_{3}+b_{24}x_{2}x_{4}+b_{34}x_{3}x_{4}+b_{22}x_{2}^{2}+b_{33}x_{3}^{2}+b_{44}x_{4}^{2}+\varepsilon$$

Given the complexity of the study, requiring the estimation of both linear and quadratic effects across variables with different factor levels (i.e., three or five levels for the quantitative continuous variables, and two levels for the qualitative categorical variable), a D-optimal design was adopted within the framework of a CCC design. Thus, from a total of 150 possible combinations (5^2^ × 3^1^ × 2^1^) (Table S1), a statistically efficient subset of 30 experiments was initially selected based on the CCC design approach (Table S2). Subsequently, applying the D-optimal criterion [55], a final reduced set of 16 experimental runs was identified (Table S3). The quality of the estimates was guaranteed since the VIFs of the coefficients of the model ranged from 1.0 to 1.3 and dmax less than 1.0 (Figures S1 and S2). To assess experimental variability between samples (inter-block) and evaluate the potential lack of fit in the model, two replicates were performed at the central point of the design space. In contrast, to assess within-sample (intra-block) variability and evaluate the homogeneity of the geopolymer specimens, the sample prepared under central point conditions for each anion type was sectioned into two portions: top and bottom. All experiments were randomized to reduce the influence of uncontrolled external factors and to prevent the introduction of bias. Experimental and D-optimal design was carried out with NEMRODW [56].

### 3.4. Geopolymer Digestion and Leaching Tests

The content of the two heavy metals stabilized in the different formulations was determined following an acid dissolution procedure. The stabilization efficiency was determined using a normalized leaching test in water.

For the geopolymer dissolution/digestion the samples were first ground into a fine powder using a vibromill (Retsch MM500 nano, Retsch GmbH, Haan, Germany) operating at 35 Hz for 2 min. This homogenized powder was used for both total metal content determination and leaching tests. For metal digestion, reverse aqua regia (3:1 HNO_3_:HCl) was used to effectively mineralize the geopolymer matrix and extract the target metals. Since the silicate framework was not of analytical interest in this study, hydrofluoric acid was not required.

Leaching tests on stabilized geopolymers were performed according to the EN 12457-2 standard test after 28 days of curing [57]. A portion of the ground geopolymer powder was mixed with deionized water at a solid-to-liquid ratio of 10 L/kg. The suspensions were agitated for 24 h using an overhead rotary shaker (Rotax 6.8, VELP Scientifica, Milan, Italy) operating at 10 rpm. After agitation, the suspensions were filtered through 0.45 µm membrane filters, and the resulting eluates were collected for analysis. Despite standard recommendations, samples were intentionally ground to a fine powder (<1 mm) in this study. This choice was justified by the specific aim of the work, which was not to assess the environmental impact of the materials but rather to evaluate the chemical immobilization of low and higher levels of metals. Fine grinding was therefore applied to minimize physical retention effects since increased surface area enhances the leaching potential and ensures that the measured leaching reflects the actual chemical stability of the immobilized species.

The solutions coming from both digested and leached samples were analyzed by Inductively Coupled Plasma Optical Emission Spectroscopy (ICP-OES) using the iCAP™ PRO X Duo system (Thermo Fisher Scientific, Waltham, MA, USA). In addition, the leaching results, that is the concentrations of total nickel and total chromium measured in the eluates, were checked with the limits established by the Italian Legislative Decree No. 121 of 3 September 2020, which implements Directive (EU) 2018/850 on the landfill of waste [58]. According to this regulation, the classification thresholds are as follows: for inert waste, 0.05 ppm for total chromium and 0.04 ppm for nickel; for non-hazardous waste, 1 ppm for both metals; and for hazardous waste, 4 ppm for nickel and 7 ppm for total chromium.

Then, the immobilization rate was calculated as follows in Equation (4) as an indicator of S/S efficiency [5]:

(4) $$immobilization\;rate\;\left(\%\right)=\;\frac{C_{G}-C_{L}}{C_{G}}.100$$

where C_L_ is the content of heavy metal leached out from the geopolymer samples, C_G_ is the total content of that metal immobilized in the geopolymer sample. The immobilization rate quantifies the percentage of metal that is effectively incorporated and stabilized within the geopolymeric network relative to the total amount introduced into the sample. Therefore, it enables the efficiency with which the material retains these specific metals to be evaluated and facilitates comparison with other formulations.

### 3.5. Characterization of Geopolymers

X-ray diffraction (XRD) analysis was carried out using D6 Phaser (Bruker, Billerica, MA, USA) equipped with a Cu-Kα radiation source to investigate the possible formation of crystalline sodalite phases. Initial diffraction patterns were recorded over the 2θ range of 5–70°, using a step size of 0.05° and a counting time of 0.2 s per step in step-scan mode. To accurately confirm the absence of zeolites, a second, more detailed scan was performed in the 10–30° range with a finer step size of 0.01° and an extended counting time of 2 s per step. The data were interpreted by comparison with the Mineral Powder Diffraction File Databook.

FT-IR spectroscopy (Prestige21 Shimadzu spectrophotometer, Shimadzu Italia s.r.l., Milan, Italy, equipped with a deuterated triglycine sulfate detector and KBr windows) was carried out on geopolymer samples. The spectra were acquired within the 4000–370 cm^−1^ range, at a resolution of 2 cm^−1^, over 60 scans. Pellets composed of 5 mg of sample and 198 mg of KBr were used for the measurements.

The multivariate analysis for FTIR spectra was performed using PLS_Toolbox [59] for use with MATLAB™ [60]. Specifically, to explore spectral differences among samples, a preliminary Principal Component Analysis (PCA) was performed. Prior to PCA, the spectral data were pre-processed using Savitzky–Golay smoothing to reduce noise (15.2), followed by conversion to absorbance (log(1/T)) and normalization with respect to the highest-intensity peak and mean-center. This normalization approach was appropriate given that the aim of the study was qualitative rather than quantitative.

## 4. Conclusions

This study presents a systematic approach to investigating the stabilization of heavy metals using a model formulation of metakaolin-based geopolymers. Using a Design of Experiments methodology, the effects and interactions of the Na/Al molar ratio, the aging time of the activating solution, the initial metal concentration and the type of anion on the leaching behavior of chromium and nickel were evaluated. The results showed that all the tested formulations were highly effective at immobilizing metals, with rates above 99.2% for both chromium and nickel. The comparison of the leachate according to EN 12457-2 standard test in water indicates that for Ni, the stabilization is more efficient than for Cr, respecting regulation limits for non-hazardous waste landfill.

This analytical and systematic approach also makes it possible to suggest possible mechanisms for metal immobilization. One novel result is the role of the aging time of the alkaline solution and the anion type. The first factor shows a strong influence on chromium leaching, especially at high concentrations. This could be because longer aging times may lead to early polymerization of the silicate solution, which reduces the capacity of the geopolymer matrix to trap chromium effectively. The second factor shows that chloride salts release more metal than sulfates.

FTIR spectroscopy supported experimental results by showing that increasing the Na/Al ratio leads to the decrease of metal leaching. This was confirmed by a shift of the main Si–O–T band toward lower wavenumbers and a reduction of the shoulder around 1100–1180 cm^−1^, which are both indicators of better network formation and structural integration of the metals.

Overall, this study supports the use of geopolymers as a sustainable and efficient solution for the treatment of metal-contaminated waste and emphasizes the importance of optimizing formulation parameters to improve performance. Future developments in the direction of the addition of Cr-chelating or absorbing agents are expected.

## Figures and Tables

**Table 1:** Summary of experimental runs, including DoE conditions (anion type, Na/Al ratio, aging time, and metal concentration), effective metal content, leaching results, and calculated immobilization rates for both chromium and nickel. The table also includes validation and homogeneity tests performed via ICP-OES on sample 9 (rep11), analyzed in top and bottom portions. In orange, the experimental matrix domain; in blue, the effective metal concentrations; in yellow, the leaching values; in green, the immobilization rates.

*n*°Exp	Run	Anion	Na/Al (mol)	Aging Time (h)	Metal (ppm)	Effective Metal (Cr) (ppm)	Effective Metal (Ni) (ppm)	Chromium Leaching (ppm)	Nickel Leaching (ppm)	Immobilization Rate (Cr) (%)	Immobilization Rate (Ni) (%)
1	3	Chloride	0.6	6	970	975	1015	1.15	0.21	98.8	99.8
2	9	Chloride	1.0	6	970	1133	1057	0.14	0.07	99.9	99.9
3	13	Chloride	1.0	24	970	1127	1117	0.14	0.08	99.9	99.9
4	2	Chloride	1.0	6	2800	3193	3367	1.51	0.28	99.5	99.9
5	6	Chloride	0.6	15	1890	2200	2183	1.67	0.59	99.2	99.7
6	1	Chloride	0.8	30	1890	1690	2113	1.04	0.10	99.4	100.0
7	14	Chloride	0.8	15	3440	3398	3094	1.37	0.39	99.6	99.9
8	11	Chloride	0.8	15	1890	2106	2106	0.36	0.10	99.8	100.0
9	rep11	Chloride	0.8	15	1890	1946	1936	0.94	0.32	99.5	99.8
10	15	Sulfate	0.6	24	970	728	1085	0.19	0.03	99.7	100.0
11	7	Sulfate	0.6	6	2800	1887	3636	0.34	0.48	99.8	99.9
12	5	Sulfate	0.6	24	2800	2169	3418	1.22	0.81	99.4	99.8
13	12	Sulfate	1.0	24	2800	1764	3046	0.19	0.11	99.9	100.0
14	16	Sulfate	0.8	15	1890	1236	2084	0.29	0.06	99.8	100.0
15	10	Sulfate	0.8	0	1890	1262	2304	0.29	0.07	99.8	100.0
16	8	Sulfate	0.8	15	340	456	516	0.15	0.03	99.7	99.9
17	4	Sulfate	0.8	15	1890	1179	1904	0.73	0.25	99.4	99.9
18	rep4	Sulfate	0.8	15	1890	1251	1936	0.20	0.07	99.8	100.0
Test point validation	17	Chloride	1.0	24	1890	2175	2320	0.51	0.03	99.8	100.0
Geopolymer homogeneity analysis (ICP-OES)											
9 (rep 11)	Top	Chloride	0.8	15	1890	1920	1892	0.98	0.33		
9 (rep 11)	Bottom	Chloride	0.8	15	1890	1972	1980	0.90	0.31		

**Table 2:** Estimates and statistics of the coefficients from the fitted response surface for nickel eluate. In bold text, the *p*-value less than α = 0.05 are relevant (i.e., the significant coefficients).

Factors	Terms	Coefficient	VIF	Standard Deviation	t Exp.	*p* Value
Intercept	b0	0.168		0.052	3.59	0.013
Anion	b1	0.052	1.20	0.033	1.74	0.152
Na/Al	b2	−0.125	1.16	0.047	−2.98	**0.028**
Aging Time (Ag.)	b3	−0.002	1.15	0.038	−0.05	0.965
Conc	b4	0.125	1.36	0.037	3.80	**0.010**
Na/Al^2^	b22	0.078	1.50	0.075	1.16	0.317
Ag. Time^2^	b33	−0.049	1.05	0.036	−1.51	0.204
Conc^2^	b44	0.017	2.03	0.040	0.46	0.683
Na/Al × Time	b23	−0.037	1.01	0.046	−0.89	0.440
Na/Al × Conc	b24	−0.064	1.16	0.038	−1.90	0.122
Ag. Time × Conc	b34	0.047	1.19	0.037	1.41	0.234

**Table 3:** Estimates and statistics of the coefficients from the fitted response surface for chrome eluate. In bold text, the *p*-value less than α = 0.05 are relevant (i.e., the significant coefficients).

Factor	Term	Coefficient	VIF	Standard Deviation	t Exp.	*p* Value
Intercept	b0	0.801		0.150	5.35	0.003
Anion	b1	0.236	1.28	0.069	3.43	**0.019**
Na/Al	b2	−0.456	2.44	0.136	−3.37	**0.020**
Aging (Ag.) Time	b3	0.311	3.10	0.120	2.60	**0.048**
Conc	b4	0.228	1.51	0.099	2.30	0.070
Na/Al^2^	b22	−0.140	1.23	0.134	−1.04	0.345
Ag.Time^2^	b33	−0.089	1.30	0.076	−1.16	0.297
Conc^2^	b44	−0.013	1.56	0.093	−0.14	0.897
Na/Al × Time	b23	0.026	1.21	0.101	0.26	0.807
Na/Al × Conc	b24	−0.022	2.55	0.189	−0.12	0.910
Ag. Time × Conc	b34	0.462	3.14	0.183	2.52	**0.053**

**Table 4:** Design of Experiments factors, coded variables, and corresponding levels used for the geopolymer formulations.

Factor	Coded Variable	Type of Variable	Levels				
			−α	−1	0	1	α
Anion	x_1_	Discrete qualitative		Sulfate		Chloride	
Na/Al (molar ratio)	x_2_	Continuous quantitative		0.6	0.8	1	
Aging time (h)	x_3_	Continuous quantitative	0	6	15	24	30
Conc. of heavy metal (ppm)	x_4_	Continuous quantitative	340	970	1890	2800	3440
α is an axian level also known as the “star point”, which is specific to composite center design (α = 1.68)							

## Data Availability

The authors can provide data upon request.

## Supplementary Materials

The following supporting information can be downloaded at: https://www.mdpi.com/article/10.3390/molecules30153235/s1, Figure S1: VIF Max versus the number of experiments. Values below the red threshold line (2.00) confirm the absence of significant multicollinearity among the variables; Figure S2: Evaluation of dMax as a fraction of the number of experiments (n). Values below the red threshold line (1.00) indicate a good model; Table S1: Matrix design of all 150 possible combinations; Table S2: CCC matrix design; Table S3: D-optimal experimental matrix. Table S4: Calculation of the differences between the nominal and effective cation contents.
