# Supplementary material for: Design of Experiments Approach for Efficient Heavy Metals Stabilization Using Metakaolin-Based Geopolymers
Source: Molecules. 2025 Aug 1;30(15):3235. doi: 10.3390/molecules30153235 (PMC12348866; doi:10.3390/molecules30153235)
Supplement: Supplementary file 1 [file molecules-30-03235-s001.zip › molecules-3736481-supplementary.pdf]

Design of Experiments Approach for Efficient Heavy Metals Stabilization Using Metakaolin-Based Geopolymers

Raffaele Emanuele Russo [1], Elisa Santoni [1], Martina Fattobene [1], Mattia Giovini [2], Francesco Genua [2], Cristina Leonelli [2], Isabella Lancellotti [2,*], Ana Herrero [3] and Mario Berrettoni [1,*]

[1] School of Science and Technology, Chemistry Division, University of Camerino, Via Madonna delle
Carceri—ChIP, 62032 Camerino, MC, Italy; raffaele.russo@unicam.it (R.E.R.);
elisa.santoni@unicam.it (E.S.); martina.fattobene@unicam.it (M.F.)
[2] Department of Engineering "Enzo Ferrari", University of Modena and Reggio Emilia, Via P. Vivarelli n. 10, 41125 Modena, MO, Italy; mattia.giovini@unimore.it (M.G.);
francesco.genua@unimore.it (F.G.);
cristina.leonelli@unimore.it (C.L.)
[3] Department of Chemistry, Faculty of Sciences, Universidad de Burgos, Plaza Misael Bañuelos s/n,
09001 Burgos, Spain; aherrero@ubu.es
* Correspondence: isabella.lancellotti@unimore.it (I.L.); mario.berrettoni@unicam.it (M.B.)

***Table S1.** Matrix Design of all 150 possible combinations*

N°Exp	Anion	Na/Al	Time	Conc
**1**	-1	-1	-1.68	-1.68
**2**	1	-1	-1.68	-1.68
**3**	-1	0	-1.68	-1.68
**4**	1	0	-1.68	-1.68
**5**	-1	1	-1.68	-1.68
**6**	1	1	-1.68	-1.68
**7**	-1	-1	-1	-1.68
**8**	1	-1	-1	-1.68
**9**	-1	0	-1	-1.68
**10**	1	0	-1	-1.68
**11**	-1	1	-1	-1.68
**12**	1	1	-1	-1.68
**13**	-1	-1	0	-1.68
**14**	1	-1	0	-1.68
**15**	-1	0	0	-1.68
**16**	1	0	0	-1.68
**17**	-1	1	0	-1.68
**18**	1	1	0	-1.68
**19**	-1	-1	1	-1.68
**20**	1	-1	1	-1.68
**21**	-1	0	1	-1.68
**22**	1	0	1	-1.68
**23**	-1	1	1	-1.68
**24**	1	1	1	-1.68
**25**	-1	-1	1.68	-1.68

**26**	1	-1	1.68	-1.68
**27**	-1	0	1.68	-1.68
**28**	1	0	1.68	-1.68
**29**	-1	1	1.68	-1.68
**30**	1	1	1.68	-1.68
**31**	-1	-1	-1.68	-1
**32**	1	-1	-1.68	-1
**33**	-1	0	-1.68	-1
**34**	1	0	-1.68	-1
**35**	-1	1	-1.68	-1
**36**	1	1	-1.68	-1
**37**	-1	-1	-1	-1
**38**	1	-1	-1	-1
**39**	-1	0	-1	-1
**40**	1	0	-1	-1
**41**	-1	1	-1	-1
**42**	1	1	-1	-1
**43**	-1	-1	0	-1
**44**	1	-1	0	-1
**45**	-1	0	0	-1
**46**	1	0	0	-1
**47**	-1	1	0	-1
**48**	1	1	0	-1
**49**	-1	-1	1	-1
**50**	1	-1	1	-1
**51**	-1	0	1	-1
**52**	1	0	1	-1
**53**	-1	1	1	-1
**54**	1	1	1	-1
**55**	-1	-1	1.68	-1
**56**	1	-1	1.68	-1
**57**	-1	0	1.68	-1
**58**	1	0	1.68	-1
**59**	-1	1	1.68	-1
**60**	1	1	1.68	-1
**61**	-1	-1	-1.68	0
**62**	1	-1	-1.68	0
**63**	-1	0	-1.68	0
**64**	1	0	-1.68	0
**65**	-1	1	-1.68	0
**66**	1	1	-1.68	0
**67**	-1	-1	-1	0
**68**	1	-1	-1	0
**69**	-1	0	-1	0
**70**	1	0	-1	0
**71**	-1	1	-1	0
**72**	1	1	-1	0

**73**	-1	-1	0	0
**74**	1	-1	0	0
**75**	-1	0	0	0
**76**	1	0	0	0
**77**	-1	1	0	0
**78**	1	1	0	0
**79**	-1	-1	1	0
**80**	1	-1	1	0
**81**	-1	0	1	0
**82**	1	0	1	0
**83**	-1	1	1	0
**84**	1	1	1	0
**85**	-1	-1	1.68	0
**86**	1	-1	1.68	0
**87**	-1	0	1.68	0
**88**	1	0	1.68	0
**89**	-1	1	1.68	0
**90**	1	1	1.68	0
**91**	-1	-1	-1.68	1
**92**	1	-1	-1.68	1
**93**	-1	0	-1.68	1
**94**	1	0	-1.68	1
**95**	-1	1	-1.68	1
**96**	1	1	-1.68	1
**97**	-1	-1	-1	1
**98**	1	-1	-1	1
**99**	-1	0	-1	1
**100**	1	0	-1	1
**101**	-1	1	-1	1
**102**	1	1	-1	1
**103**	-1	-1	0	1
**104**	1	-1	0	1
**105**	-1	0	0	1
**106**	1	0	0	1
**107**	-1	1	0	1
**108**	1	1	0	1
**109**	-1	-1	1	1
**110**	1	-1	1	1
**111**	-1	0	1	1
**112**	1	0	1	1
**113**	-1	1	1	1
**114**	1	1	1	1
**115**	-1	-1	1.68	1
**116**	1	-1	1.68	1
**117**	-1	0	1.68	1
**118**	1	0	1.68	1
**119**	-1	1	1.68	1

**120**	1	1	1.68	1
**121**	-1	-1	-1.68	1.68
**122**	1	-1	-1.68	1.68
**123**	-1	0	-1.68	1.68
**124**	1	0	-1.68	1.68
**125**	-1	1	-1.68	1.68
**126**	1	1	-1.68	1.68
**127**	-1	-1	-1	1.68
**128**	1	-1	-1	1.68
**129**	-1	0	-1	1.68
**130**	1	0	-1	1.68
**131**	-1	1	-1	1.68
**132**	1	1	-1	1.68
**133**	-1	-1	0	1.68
**134**	1	-1	0	1.68
**135**	-1	0	0	1.68
**136**	1	0	0	1.68
**137**	-1	1	0	1.68
**138**	1	1	0	1.68
**139**	-1	-1	1	1.68
**140**	1	-1	1	1.68
**141**	-1	0	1	1.68
**142**	1	0	1	1.68
**143**	-1	1	1	1.68
**144**	1	1	1	1.68
**145**	-1	-1	1.68	1.68
**146**	1	-1	1.68	1.68
**147**	-1	0	1.68	1.68
**148**	1	0	1.68	1.68
**149**	-1	1	1.68	1.68
**150**	1	1	1.68	1.68

Table S2. *CCC matrix design*

N°Exp	Run	Anion	Na/Al	Time	Conc
			Unit2	Unit3	Unit4
**1**		1	-1	-1	-1
**2**		1	1	-1	-1
**3**		1	-1	1	-1
**4**		1	1	1	-1
**5**		1	-1	-1	1
**6**		1	1	-1	1
**7**		1	-1	1	1
**8**		1	1	1	1
**9**		1	-1.6818	0	0

**10**		1	1.6818	0	0
**11**		1	0	-1.6818	0
**12**		1	0	1.6818	0
**13**		1	0	0	-1.6818
**14**		1	0	0	1.6818
**15**		1	0	0	0
**16**		-1	-1	-1	-1
**17**		-1	1	-1	-1
**18**		-1	-1	1	-1
**19**		-1	1	1	-1
**20**		-1	-1	-1	1
**21**		-1	1	-1	1
**22**		-1	-1	1	1
**23**		-1	1	1	1
**24**		-1	-1.6818	0	0
**25**		-1	1.6818	0	0
**26**		-1	0	-1.6818	0
**27**		-1	0	1.6818	0
**28**		-1	0	0	-1.6818
**29**		-1	0	0	1.6818
**30**		-1	0	0	0

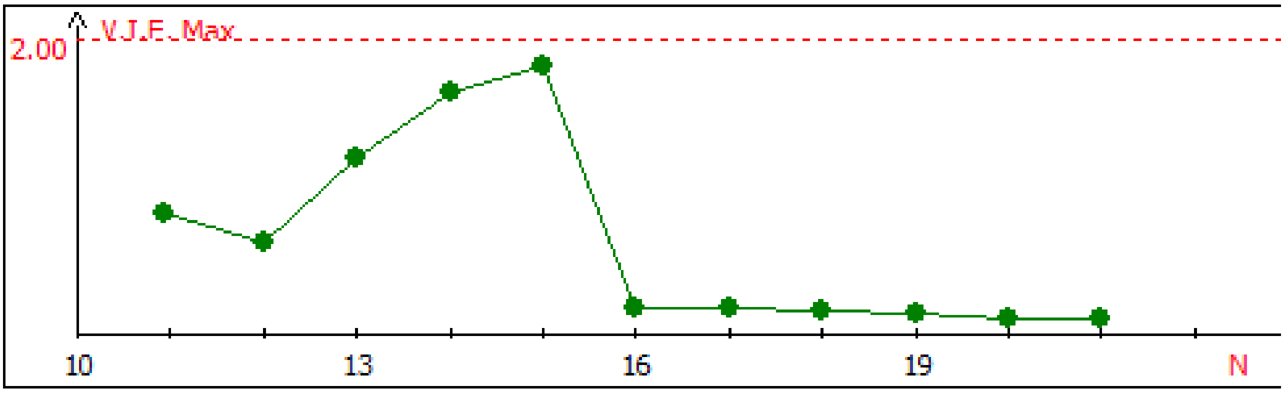

Figure S1. *VIF Max versus the number of experiments. Values below the red threshold line (2.00) confirm the absence of significant multicollinearity among the variables.*

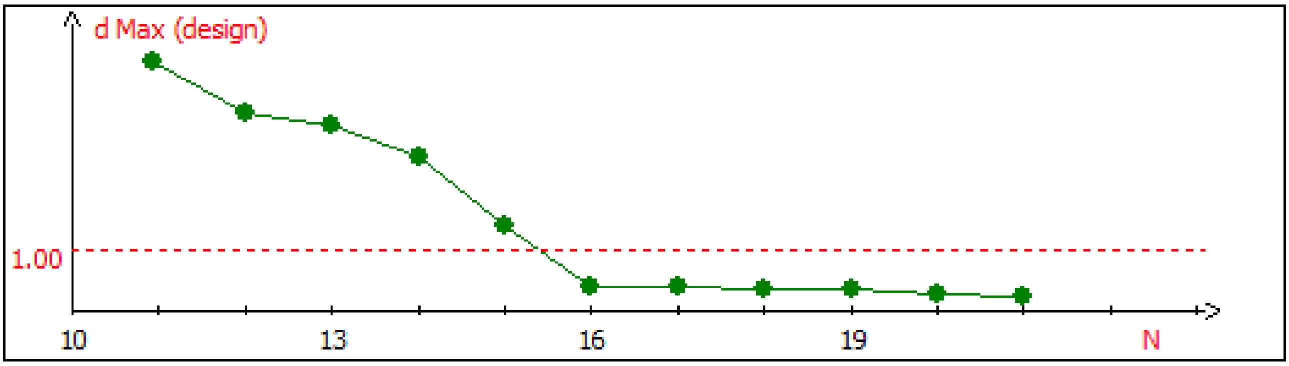

Figure S2. *Evolution of the dMax as a function of the number of experiments (N). Values below the red threshold line (1.00) indicate a good model.*

Table S3. *D-Optimal experimental matrix*

N°Exp	Run	Anion	Na/Al	Time	Conc
**1**	3	1	-1	-1	-1
**2**	9	1	1	-1	-1
**3**	13	1	1	1	-1
**4**	2	1	1	-1	1
**5**	6	1	-1	0	0
**6**	1	1	0	1.68179	0
**7**	14	1	0	0	1.68179
**8**	**11**	1	0	0	0
**9**	15	-1	-1	1	-1
**10**	7	-1	-1	-1	1
**11**	5	-1	-1	1	1
**12**	12	-1	1	1	1
**13**	16	-1	1	0	0
**14**	10	-1	0	-1.68179	0
**15**	8	-1	0	0	-1.68179
**16**	**4**	-1	0	0	0

Table S4. *Calculation of the differences between the nominal and effective cation contents.*

N°Exp	Run	Anion	Na/Al (mol)	ageing time (h)	Metal (ppm)	Effective metal (Cr) (ppm)	error between Cr theoretical and effective quantity %	Effective metal (Ni) (ppm)	error between Ni theoretical and effective quantity %
1	3	Chloride	0.6	6	970	975	**0.52**	1015	**4.64**
2	9	Chloride	1	6	970	1133	**16.80**	1057	**8.97**
3	13	Chloride	1	24	970	1127	**16.19**	1117	**15.15**
4	2	Chloride	1	6	2800	3193	**14.04**	3367	**20.25**
5	6	Chloride	0.6	15	1890	2200	**16.40**	2183	**15.50**
6	1	Chloride	0.8	30	1890	1690	**-10.58**	2113	**11.80**
7	14	Chloride	0.8	15	3440	3398	**-1.22**	3094	**-10.06**
8	11	Chloride	0.8	15	1890	2106	**11.43**	2106	**11.43**
9	rep11	Chloride	0.8	15	1890	1946	**2.96**	1936	**2.43**
10	15	Sulphate	0.6	24	970	728	**-24.95**	1085	**11.86**
11	7	Sulphate	0.6	6	2800	1887	**-32.61**	3636	**29.86**
12	5	Sulphate	0.6	24	2800	2169	**-22.54**	3418	**22.07**
13	12	Sulphate	1	24	2800	1764	**-37.00**	3046	**8.79**
14	16	Sulphate	0.8	15	1890	1236	**-34.60**	2084	**10.26**
15	10	Sulphate	0.8	0	1890	1262	**-33.23**	2304	**21.90**
16	8	Sulphate	0.8	15	340	456	**34.12**	516	**51.76**
17	4	Sulphate	0.8	15	1890	1179	**-37.62**	1904	**0.74**
18	rep4	Sulphate	0.8	15	1890	1251	**-33.81**	1936	**2.43**
